# The Evaluation of a Web-Based Intervention (Deprexis) to Decrease Depression and Restore Functioning in Veterans: Protocol for a Randomized Controlled Trial

**DOI:** 10.2196/59119

**Published:** 2024-10-24

**Authors:** Rahel Pearson, Christopher G Beevers, Joseph Mignogna, Justin Benzer, Paul N Pfeiffer, Edward Post, Suzannah K Creech

**Affiliations:** 1 Veterans Integrated Service Network 17 Center of Excellence for Research on Returning War Veterans Central Texas Veterans Affairs Healthcare System Waco, TX United States; 2 Department of Psychology and Institute for Mental Health Research University of Texas at Austin Austin, TX United States; 3 Rocky Mountain Mental Illness, Research, Education and Clinical Center for Suicide Prevention Rocky Mountain Regional VHA Medical Center Aurora, CO United States; 4 Department of Physical Medicine and Rehabilitation University of Colorado Anschutz Medical Campus Aurora, CO United States; 5 Department of Psychiatry and Behavioral Sciences Dell Medical School University of Texas at Austin Austin, TX United States; 6 Veterans Affairs Ann Arbor Healthcare System Ann Arbor, MI United States; 7 Department of Psychiatry University of Michigan Medical School Ann Arbor, MI United States

**Keywords:** depression, eHealth, mental health, randomized controlled trial, RCT, mobile phone

## Abstract

**Background:**

Depressive symptoms are common in veterans, and the presence of these symptoms increases disability as well as suicidal thoughts and behaviors. However, there is evidence that these symptoms often go untreated. Intervening before symptoms become severe and entrenched is related to better long-term outcomes, including improved functioning and less disease chronicity. Computer-delivered interventions may be especially appropriate for those veterans with mild to moderate depressive symptoms, because these interventions can require fewer resources and have lower barriers to access and thus have potential for wider reach. Despite this potential, there is a dearth of research examining computerized interventions for depressive symptoms in veteran samples.

**Objective:**

The aim of this study is to evaluate the efficacy of Deprexis (GAIA AG), a computerized intervention for depressive symptoms and related functional impairment.

**Methods:**

Veterans will be recruited through the US Department of Veterans Affairs electronic medical record and through primary care and specialty clinics. First, qualitative interviews will be completed with a small subset of veterans (n=16-20) to assess the acceptability of treatment procedures. Next, veterans (n=132) with mild to moderate depressive symptoms will be randomly assigned to the fully automated Deprexis intervention or a treatment-as-usual control group. The primary outcomes will be self-reported depressive symptoms and various dimensions of psychosocial functioning.

**Results:**

This project was funded in May 2024, and data collection will be conducted between October 2024 and April 2029. Overall, 4 participants have been recruited as of the submission of the manuscript, and data analysis is expected in June 2029, with initial results expected in November 2029.

**Conclusions:**

This study will provide initial evidence for the efficacy of self-guided, computerized interventions for depressive symptoms and functional impairment in veterans. If effective, these types of interventions could improve veteran access to low-resource psychosocial treatments.

**Trial Registration:**

ClinicalTrials.gov NCT06217198; https://www.clinicaltrials.gov/study/NCT06217198

**International Registered Report Identifier (IRRID):**

PRR1-10.2196/59119

## Introduction

### Background and Rationale

Depression is the leading cause of disability worldwide [1], and those diagnosed with major depressive disorder (MDD) are up to 7.6 times more likely to die by suicide compared to the general population [2]. Depressive symptoms are associated with severe and chronic impairment in occupational and social domains [3], decreased quality of life [4], and adverse physical health outcomes [5]. Depression is a significant problem for US military veterans, with 13% of post-9/11 war veterans meeting criteria for current MDD [6] and 46.5% of female veterans and 36.3% of male veterans meeting criteria for lifetime MDD [7]. The high prevalence of depressive symptoms in veterans not only has negative ramifications for social and interpersonal functioning, occupational participation, and quality of life [8] but also markedly increases the risk for suicide. On average, 543 veterans die by suicide each month [9], and MDD is the second most important predictor of veteran suicide among all recorded mental health diagnoses [10]. Depressive comorbidity among veterans with posttraumatic stress disorder (PTSD) compared to PTSD alone is also associated with elevated rates of disability in cognitive (Cohen *d*=1.03) and mental health (Cohen *d*=1.49) domains. Veterans with comorbid PTSD and MDD also reported decreases in quality of life (Cohen *d*=0.84) and a 2-fold risk of suicide compared to those with PTSD alone [11]. Given the functional impairments and elevated suicidal risk associated with depressive symptoms, it is essential to provide veterans with timely and appropriate intervention.

Addressing depression before the symptoms become severe and entrenched could substantially improve outcomes. Even mild to moderate and subthreshold depressive symptoms are associated with significant disease burden, including increases in disability and mortality and decreases in quality of life [12]. The rates of transition to MDD for individuals with subthreshold depressive symptoms are exceedingly high both in the short term [13] and long term [14]. When subthreshold symptoms progress to MDD, symptoms become chronic in the vast majority of cases, with 85% of individuals with MDD experiencing recurrent episodes even after receiving specialty mental health treatment [15]. Evidence suggests that interventions targeted at individuals with mild to moderate symptoms, including those with subthreshold symptoms of MDD, are effective in restoring functioning and preventing the progression to severe disease [16].

Although depressive symptoms often go undetected at mild to moderate or subthreshold levels in community settings [17], depression symptom screenings are routinely performed on a population level at US Department of Veterans Affairs (VA) facilities. This provides a key opportunity to identify veterans with depressive symptoms, even before these symptoms reach the diagnostic threshold, allowing for rapid and high-quality intervention. Patient preference studies indicate that individuals with depressive symptoms prefer to receive psychotherapy over medication [18]. Although the VA has made significant strides in improving access to psychotherapy, including through the expansion of primary care–mental health integration [19], many veterans still struggle to access adequate psychotherapy. Research shows that the majority of veterans (61%) with diagnosed mental health disorders only attend 1 session of psychotherapy [20]. Shame and stigma around depressive symptoms and seeking mental health treatment, as well as difficulties attending mental health treatments due to a lack of time or transportation, are known barriers to adequate and timely intervention and care [21]. Although the expansion of virtual mental health care (ie, telehealth) within VA facilities is effective at overcoming certain logistical barriers to treatment, telehealth is as resource intensive as traditional face-to-face psychotherapy, and access is dependent on provider availability. Furthermore, VA telehealth services might be less appropriate for individuals with mild to moderate symptoms, whose symptom severity level might not merit individual psychotherapy, but who would nonetheless benefit from help. Thus, despite increased detection of depressive symptoms and an identified need for intervention, a significant treatment gap exists.

Internet-delivered interventions are well positioned to fill this treatment gap because these interventions are low cost, easily accessible, and scalable. When compared to telehealth or traditional face-to-face mental health care, internet-delivered interventions could alleviate veterans’ stigma concerns and provide increased flexibility of access in the presence of competing demands of work and childcare. Positive experiences with internet-delivered interventions could also facilitate the eventual transition to traditional mental health care if needed [22]. Meta-analyses of results from community samples provide evidence for the effectiveness of internet-delivered interventions for depressive symptoms, including acceptance and commitment therapy [23], cognitive behavioral therapy [24,25], and mindfulness-based approaches [26].

Deprexis (GAIA AG) [27,28] is an internet-delivered intervention that integrates acceptance and commitment therapy, cognitive behavioral therapy, and psychodynamic and mindfulness-based approaches. The program consists of 10 core modules targeting depressive symptoms and associated functional impairment. Engagement with content is completely self-guided, and the program is accessed remotely. A randomized controlled trial (RCT) of Deprexis in a general population sample found that it was effective in reducing depressive symptoms, improving well-being, and decreasing disability [29]. Despite these positive results in the general population, it is unknown whether Deprexis is an effective treatment for veterans with depressive symptoms, given the unique composition of the veteran population in terms of gender ratio, barriers to treatment, the severity of comorbid conditions, and sociodemographic characteristics. Furthermore, there is a need to understand whether this low-intensity, internet-delivered treatment for depression also addresses functional impairments in veterans. A critical gap in the field is testing the effects of evidence-based mental health interventions on recovery outcomes. The primary goal in previous trials was to test the effect of Deprexis on depressive symptoms. Although improvements in functional domains after Deprexis treatment have been reported [29], the conducted assessments did not span the full range of functional outcomes. There is a need to centralize the assessment of psychosocial functioning in mental health delivery research generally and the study of Deprexis specifically.

### Aims

Deprexis has not yet been rigorously evaluated with a veteran population in a VA medical center setting. This study aims to fill this critical gap in the literature by testing whether Deprexis is acceptable and effectively decreases depressive symptoms and improves functional outcomes in veterans (N=152) presenting for VA health care with mild to moderate depressive symptoms. The objective of aim 1 is to assess perceptions, needs, and preferences as they relate to Deprexis through conducting posttreatment assessment interviews with veterans (n=16-20) and identify the components that improve veterans’ experience and uptake of Deprexis. The results obtained in aim 1 will be used to inform the rollout of RCT procedures under aim 2. Aim 2a involves completing an RCT comparing an 8-week course of Deprexis to a treatment-as-usual (TAU) control condition. We hypothesize that veterans engaged in Deprexis will show improvements on measures of functioning and decreases in depressive symptoms compared to the TAU control group. To generate hypotheses for future study, the objective of exploratory aim 2b will be to examine whether demographic variables, baseline psychopathology, credibility, and Deprexis use moderate treatment effects on the primary outcomes.

## Methods

### Trial Design

This study is an individually randomized, parallel, 2-group therapy trial. A total of 132 veterans will be randomized in a 1:1 fashion to either a Deprexis intervention or a TAU control group.

### Study Setting

Recruitment will take place within the VA Central Texas Healthcare System. All assessment and intervention components will be completed remotely through Qualtrics (Qualtrics International Inc) [30] and the Deprexis website, respectively.

### Eligibility Criteria

Potential participants include all veterans irrespective of sex or gender and of all ages and races and ethnicities who (1) are able to comprehend and sign the informed consent form; (2) have reliable access to the internet and a computer, tablet device, or smartphone; (3) exhibit mild to moderate (not very severe) levels of depression (Quick Inventory of Depressive Symptomatology–Self-Report [QIDS-SR] [31] score between 6 and 20 at the time of screening); and (4) are stable on psychotropic medications (defined as no medication change in the 30 days before study entry). The last criterion is included to ensure that the symptoms evaluated during the pretreatment assessment are due to underlying psychiatric conditions and not because of starting or stopping medications. Veterans will be excluded from study participation if they (1) endorse any positive symptoms of a psychotic disorder (assessed via the Psychiatric Diagnostic Screening Questionnaire [PDSQ] [32]), (2) screen positive for bipolar I disorder (assessed via the Mood Disorder Questionnaire [33]), or (3) report current suicidal risk (assessed via Beck Depression Inventory–Second Edition [BDI-II] item 9 [34]).

### Recruitment

For this study, veterans presenting with mild to moderate symptoms of depression will be recruited. Recruitment letters, including a link to the Qualtrics survey, will be sent out by US mail to veterans identified through the medical record who screen positive on the Patient Health Questionnaire (PHQ)-2 clinical reminder screen (a score of ≥3). The initial eligibility criterion is purposefully broad (ie, a positive PHQ-2 screen vs a positive PHQ-9 screen) because we do not want to exclude treatment-receptive veterans with mild depressive symptoms. Veterans’ telephone numbers will be obtained from the medical record, and a research technician will contact veterans by telephone to ensure that they have received their letters, answer any questions, and provide them with a link to the Qualtrics survey if needed. Flyers at VA primary care and medical specialty clinics will also be posted and distributed, and information about the study and participant inclusion and exclusion criteria will be provided to VA primary care providers.

### Intervention Description

**Figure 1:**
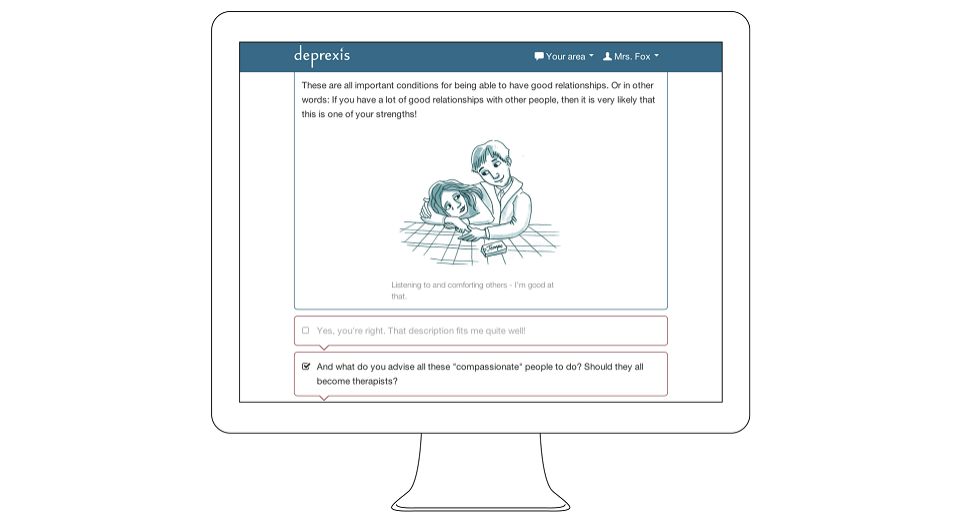
Deprexis sample presentation.

Deprexis is an internet-delivered treatment for depressive symptoms and related functional impairment. The intervention draws from various theoretical frameworks and consists of 12 modules: 10 core content modules, an introductory module, and a summary module. Deprexis, which is designed to be interactive involves answering questions and learning techniques and concepts through instruction and examples. Deprexis uses a software technology (Broca) that tailors content dynamically to the users’ responses, resulting in a simulated conversational flow. To increase motivation, Deprexis features symptom tracking, with accompanying graphical and text feedback, worksheets and summaries in printable format, audio recordings, and illustrations. The content focuses on improving depressive symptoms (eg, cognitive restructuring of depressogenic thoughts) and decreasing functional deficits associated with depressive symptoms (eg, behavioral activation, interpersonal skills, and healthy lifestyle choices). The program is self-paced, and every module takes between 10 and 60 minutes to complete. Participants are provided a free Deprexis voucher and will access the intervention through the Deprexis website. Textbox 1 [27] presents an overview of session content, while Figure 1 depicts a Deprexis sample presentation.

**Deprexis module content.**

**Introduction**

- Overview of the Deprexis program; psycho-education about the relationships among thoughts, feelings, and behaviors; observing and accepting thoughts and feelings; mindfulness exercise; a worksheet to encourage regular program use

**Behavioral activation**

- Exploring the relationship between activity and depressive symptoms; psycho-education on basic psychological needs (eg, need for competence and social relatedness); selecting and scheduling activities that satisfy basic psychological needs using a checklist and log; exploring and problem-solving barriers to completing activities

**Cognitive modification**

- Psycho-education on automatic thoughts; exploring antecedents and consequences of automatic thoughts; common cognitive distortions; strategies for correcting cognitive distortions (eg, birds-eye view and a scientist’s perspective)

**Relaxation, physical exercise, and lifestyle modification**

- Relationship between lifestyle habits (eg, sleep, exercise, and diet) and depression; instruction and practice of various relaxation exercises to help mitigate stress (eg, diaphragmatic breathing and visual imagery)

**Acceptance and mindfulness**

- Illustrate the difficulty of controlling thoughts and feelings and the alternative of calmly accepting unwanted thoughts and feelings using metaphors and exercises; mindfulness exercises that promote acceptance (eg, leaves on a stream); clarifying values and taking value-consistent action

**Problem solving**

- Strengthening problem-solving skills by defining problems in concrete rather than vague terms, setting achievable goals, generating a variety of potential solutions, evaluating solutions, implementing the chosen solution, and evaluating outcomes

**Childhood experiences**

- Exploring difficult childhood memories and using coping skills such as expressive writing, acceptance, forming new positive memories, and forgiveness

**Interpersonal skills**

- Psycho-education about the relationship between interpersonal functioning and depression; exploring different communication styles (eg, assertive, passive-aggressive, aggressive, and nonverbal communication); practicing communication techniques (eg, nonblaming communication)

**Positive psychology**

- Psycho-education about positive psychology; focusing on strengths instead of deficits; fostering happiness by savoring positive experiences and identifying and fostering talents

**Dreamwork and an emotion-focused intervention (optional)**

- Coping with distressing dreams (eg, keeping a dream journal and rewriting the ending of dreams); reconceptualizing dreams as a way for our brain to help us problem solve

Veterans randomized to the control condition will have access to standard VA treatment resources, including mental health treatment (they will be provided with a list of available resources).

### Criteria for Discontinuing or Modifying Allocated Interventions

Veterans can choose to discontinue study involvement at any time without facing any consequences. We will actively monitor for any serious adverse events occurring during their study involvement. Modifications to intervention components or procedures may be made at the discretion of the principal investigator (PI; RP), pending approval from the institutional review board (IRB) and other relevant stakeholders.

### Strategies to Improve Adherence to Intervention Protocols

Adherence will be optimized using strategies from previous Deprexis trials. All veterans will be sent brief SMS text messages to increase engagement. These SMS text messages will target behavioral and cognitive processes that are known to maintain depressive symptoms and will include reminders to use the Deprexis program. Research staff will also have access to veterans’ real-time Deprexis use data, and based on this use data, short encouraging emails will be sent to them by research staff. These emails will range from acknowledging the veterans’ use of the Deprexis program to encouraging further engagement and addressing feelings of discouragement. Research staff will also reach out to veterans who have not activated their free Deprexis vouchers to provide technical support if needed. Study staff will initiate contact with veterans if there is no engagement with Deprexis over a 2-week period.

### Relevant Concomitant Care and Interventions That Are Permitted or Prohibited During the Trial

To participate, veterans need to be stable (no changes in the last 30 days) on medications prescribed for a mental health disorder. Veterans randomized to either treatment group are allowed to start or continue any nonstudy treatment resources, including mental health treatment, during their participation in the trial.

### Participant Timeline

**Figure 2:**
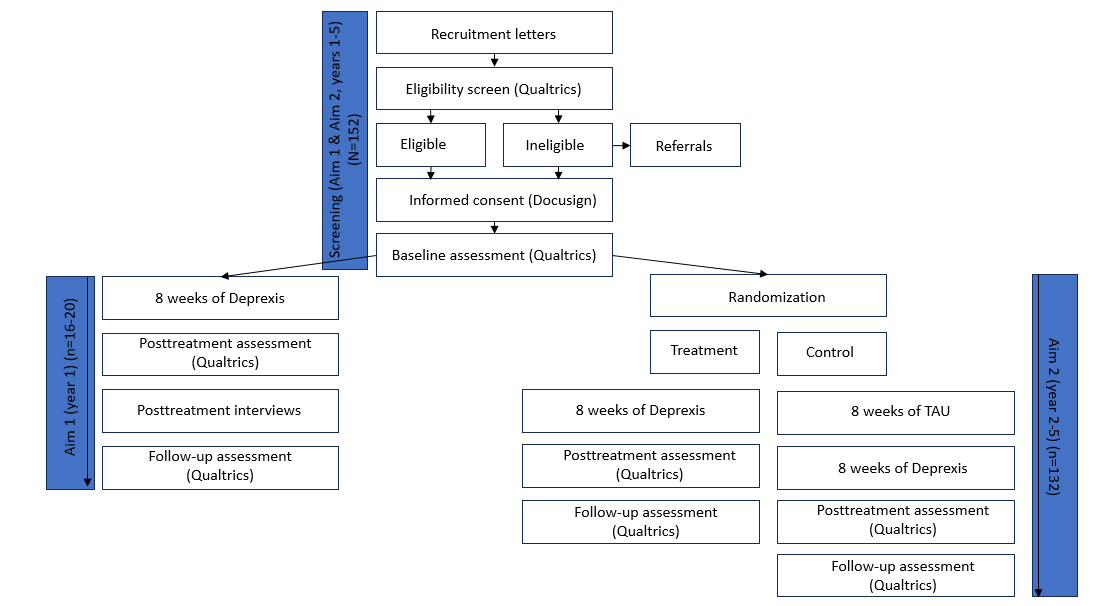
Participant timeline.

Figure 2 shows the participant timeline in detail.

### Outcomes

In aim 1, qualitative veteran feedback on the Deprexis intervention and study procedures will be obtained; no quantitative analyses will be completed. Broadly, the posttreatment interview questions will cover the following content areas: (1) motivation toward, and barriers to, treatment and participation in the study; (2) Deprexis content, presentation, adherence, and usability; (3) perceptions of study procedures; and (4) changes experienced in mood, behavioral engagement, or interpersonal interactions after Deprexis treatment.

In aim 2a, we will test whether Deprexis is effective for decreasing depressive symptoms and improving functional outcomes in veterans presenting for VA health care with mild to moderate depressive symptoms. The primary outcomes for this aim are depressive symptoms (assessed with the QIDS-SR-16) and functioning (assessed with the World Health Organization Disability Assessment Schedule II [WHODAS-II] and the Sheehan Disability Scale [SDS]). The secondary outcomes are other psychopathology (assessed with the PTSD Checklist for Diagnostic and Statistical Manual of Mental Disorders, Fifth Edition [PCL-5] and the PDSQ), social functioning (assessed with the Interpersonal Support Evaluation List–Short Form [ISEL-SF] and the Inventory of Interpersonal Problems–Short Circumplex Form [IIP-SC]), and quality of life (assessed with the Quality of Life Scale [QLS]).

Exploratory analyses in aim 2b will examine whether demographic variables (sex, gender, age, race, ethnicity, and education), PTSD symptom severity (assessed with the PCL-5), comorbidity (assessed with the PDSQ), treatment credibility (assessed with the Credibility and Expectancy Questionnaire), frequency of study support, and Deprexis use moderate treatment effects.

### Measures

The following primary outcomes (assessed at all time points) are administered through Qualtrics:

The QIDS-SR-16 [31] is a 16-item self-report measure of depressive symptom severity. This measure takes 5 to 7 minutes to complete and assesses the 9 Diagnostic and Statistical Manual of Mental Disorders, Fourth Edition (DSM-V), symptom criterion domains for depression (eg, “There is no change in my usual appetite”) and has been shown to be highly internally consistent (Cronbach α=0.86). The QIDS-SR-16 demonstrates concurrent validity with measures assessing similar outcomes (*r*=0.86-0.65) and is sensitive to symptom change [31]. Scores of >6 are indicative of meaningful depressive symptoms. The QIDS-SR-16 will serve as a primary eligibility criterion, specifically a QIDS-SR-16 score between 6 and 20. To verify the duration of depressive symptoms, we will include an item adapted from the Clinical Interview Schedule–Revised, which will assess the duration of depressive symptoms on a 6-point scale.

The WHODAS-II [35] is a 36-item self-report questionnaire, which takes 5 to 20 minutes to complete. The measure assesses functional disability across 7 domains: understanding and communicating, getting around, getting along with people, life activities, work, participation in society, and self-care (eg, “In the last 30 days, how much difficulty did you have in concentrating and doing something for 10 minutes?”). The WHODAS-II total score is scaled from 0 to 100, with higher scores indicating greater disability. The WHODAS-II has high test-retest reliability (*r*=0.98) and moderate to good concurrent validity with measures assessing similar outcomes (*r*=0.45-0.65), and it is sensitive to change in functional disability after treatment [35].

The SDS [36] is a 3-item self-report measure of symptom-related disability (eg, “The symptoms have disrupted your work/school life”) that has been used in previous Deprexis trials. The SDS takes approximately 5 minutes to complete, and items are summed (range 0-30), with higher scores indicating greater disability. The SDS was developed as a global measure of the impact of mental illness on work and school activities, family relationships, and social functioning. The SDS is highly internally consistent (Cronbach α=0.89) and has good reliability (*r*=0.73and high construct validity [37]; in addition, it is sensitive to treatment effects [38].

The secondary outcomes listed in the following sections are assessed at all time points.

The PDSQ [32] is a 125-item questionnaire containing 13 subscales (eg, MDD, anxiety disorders, and alcohol and drug abuse and dependence) that is used to screen for symptoms of mental health disorders. The PDSQ takes 15 to 20 minutes to complete, with scoring and cutoffs varying by individual scale [34]. This scale shows high internal consistency (Cronbach α=0.82); high test-retest reliability (*r*=0.84); and good discriminant, convergent (mean *r*=0.17), and concurrent (mean *r*=0.72) validity [32].

The BDI-II [34] is a 21-item self-report measure assessing depression symptom severity, completed in approximately 10 minutes. Items are scored on a 4-point scale (range 0-63), with higher scores reflecting increased endorsement of depressive symptoms. A score of ≥15 indicates the presence of clinically significant depressive symptoms [39]. The BDI-II has high internal consistency (Cronbach α=0.90), good to high test-retest reliability (*r*=0.73-0.96), and it correlates highly with interview-based measures of depression (*r*=0.66-0.75) [40]. The BDI-II will be used as a secondary measure of depression and to assess the suicidal ideation exclusion criteria.

The IIP-SC [41] is a 32-item self-report measure assessing the interference of depressive thoughts, behaviors, and symptoms across various psychosocial domains (eg, “It is hard for me to join in on groups”). The measure takes 10 to 15 minutes to complete. It consists of 8 scales consisting of 4 items each. Scores are averaged (range 0-4), with higher scores indicating greater interpersonal problems. The IIP-SC is sensitive to treatment effects and has demonstrated good to high internal consistency (Cronbach α=0.64-0.87) and good to high test-retest reliability (*r*=0.56-0.81) [42].

The ISEL-SF [43] is a 12-item self-report measure assessing perceived social support across four subscales: (1) appraisal support (the perceived availability of someone to discuss issues of personal importance), (2) tangible assets support (the perceived availability of material aid), (3) belonging support (the perceived availability of others to interact with socially), and (4) self-esteem support (the perceived availability of others with whom one compares favorably). The measure takes approximately 5 minutes to complete, with higher scores indicating the presence of increased social support. There is evidence that perceived social support improves after internet-delivered interventions for depression [44]. The ISEL-SF has been found to be highly internally consistent (Cronbach α=0.83) and has demonstrated moderate convergent construct validity with other measures of social support (*r*=0.45) [45].

The QLS [46] is a 16-item self-report measure of quality of life across various domains, including material and physical well-being; relationships; social, community, recreational, and civic activities; personal development; and fulfillment and independence. Ratings for domains (eg, “Health, being physically fit, and vigorous”) are assigned on a 7-point scale from “delighted” to “terrible” (range 12-116), with higher scores indicating a better quality of life. The measure takes approximately 10 minutes to complete. The QLS has high to excellent internal consistency (Cronbach α=0.82-0.92) and high test-retest reliability (*r*=0.78-0.84), and it has demonstrated good convergent validity with similar measures (*r*=0.67-0.75) [47]. Furthermore, the QLS is able to discriminate between populations with expected differences in quality of life (ie, those with chronic illness and those without) [48].

The PCL-5 [49] will be used to identify whether PTSD is a potential moderator of symptom change and to examine whether Deprexis produces meaningful changes in PTSD symptoms. The version of the PCL-5 used (PCL-5 with Criterion A) will include an assessment of the presence of a traumatic event meeting criterion A (the first criterion for diagnosing PTSD according to the Diagnostic and Statistical Manual of Mental Disorders, Fifth Edition). If no traumatic event is endorsed, veterans will not complete the remaining PCL-5 questions. Veterans who endorse exposure to a criterion A traumatic event will complete the 20-item PCL-5, which assesses PTSD symptom severity on a 5-point scale (eg, “In the past month, how much were you bothered by ‘Repeated, disturbing, and unwanted memories of the stressful experience?’”). Scores range from 0 to 80, with scores of >31 being indicative of probable PTSD (ref). The measure takes approximately 10 minutes to complete. The measure has excellent internal consistency (Cronbach α=0.94), high test-retest reliability (*r*=0.82) [49], and moderate convergent and discriminant validity (*r*=0.31-0.60) [49]. The PCL-5 is as sensitive to clinical change that occurs between pre- and posttreatment assessments as gold standard interview-based measures of PTSD symptoms (alerting convergent validity: *r*=0.94 and contrast converting validity: *r*=0.92) [50].

The following moderators and covariates are measured at baseline:

1. The Credibility and Expectancy Questionnaire [51] is a 6-item self-report survey used to measure participants’ perceived treatment expectancy and the credibility of clinical outcome studies (eg, “At this point, how logical does the course offered to you seem?”). The measure takes approximately 5 minutes to complete, with higher scores indicating increased treatment credibility. Treatment credibility predicts therapy outcome [52] and was an important predictor of Deprexis treatment response in the general population RCT [53]. The measure has high internal consistency (Cronbach α=0.84) and high test-retest reliability (*r*=0.83) [52].
2. The Client Satisfaction Questionnaire-8 [54] will be administered after treatment to assess the quality of services, treatment satisfaction, and willingness to recommend the treatment to others (eg, “How would you rate the quality of service that you received?”). The self-report measure takes approximately 5 minutes to complete and consists of 8 items scored on a 4-point scale (range 4-32), with higher scores representing greater acceptability. The measure has high internal consistency (Cronbach α=0.91), and it has been shown to correlate with treatment attendance (*r*=0.54) and outcomes (*r*=–0.35) [55].
3. Participants will self-report on demographic variables, including sex, gender, race, age, ethnicity, marital status, employment, and income. The demographic questionnaire will take approximately 10 minutes to complete.

### Sample Size

#### Aim 1

In determining the sample size for qualitative studies, the key goals are to develop case-oriented perspectives, select participants with a range of characteristics and perspectives, and achieve saturation (ie, the point at which subsequent interviews fail to produce new themes) [56]. Research indicates that typically, saturation is reached with 12 to 16 interviews. As we are also obtaining qualitative data on veterans who did not adhere to the treatment (approximately 23% of the sample), we increased our recruitment target slightly to 16 to 20 veterans to allow for a sufficient sample of veterans who completed Deprexis treatment, as well as those who terminated treatment early (ie, did not complete the posttreatment or follow-up questionnaires).

#### Aim 2

Effect sizes in the general population Deprexis RCT for participants with mild to moderate depression (QIDS-SR-16 score <21) ranged from Cohen *d*=0.65 to Cohen *d*=0.90. Some evidence suggests that effect sizes for psychological interventions may be smaller for veterans [57]; therefore, we estimate that effect sizes are at least medium (Cohen *d*=0.50) for measures of functioning and large (Cohen *d*=0.80) for depressive symptoms. To achieve 0.8 power at an α of .05, a total of 102 participants need to be recruited. Accounting for 23% attrition over time, we will randomize 132 participants at baseline. In the general population trial, approximately 57% (n=213) of the randomized participants were high engagers (>90 min), and effect sizes were slightly higher in the high engager group (Cohen *d*>0.67) than in the overall sample. Thus, we expect to be adequately powered for analysis in this subgroup.

### Assignment of Intervention: Allocation and Blinding

#### Allocation Sequence Generation

Computer-generated stratified block randomization with block sizes of 4 and 8 will be performed by the research technician using a web-based application. As depression severity has previously been associated with Deprexis treatment response, randomization will be stratified by depression severity, consisting of 3 categories: mild=QIDS-SR-16 score 6-10, mild to moderate=QIDS-SR-16 score 11-15, and moderate=QIDS-SR-16 score <16-20. Stratification was limited to depression severity because minimizing the number of strata is recommended in trials that enroll relatively few participants.

#### Who Will Be Blinded?

The use of a TAU control condition precludes the blinding of participants and research staff. Confounds due to research staff not being blinded will be minimal because all assessment and intervention components will be completed remotely without research staff support. Given that we only use self-report measures, the involvement of research staff in responses will be very limited, and rater effects are unlikely.

### Data Collection, Management, and Analysis

#### Plans to Promote Participant Retention

Participants will receive SMS text and email reminders with embedded links to their Qualtrics follow-up assessments. Research technicians will attempt to make telephone contact with veterans who fail to complete follow-up assessments within the designated time frame.

#### Data Management

Various steps will be taken to ensure data quality. First, questionnaire data will be directly downloaded from Qualtrics into a Microsoft Excel file. Statistical software code will then be used to score the questionnaires. Two different research technicians will double score the questionnaires, and any discrepancies will be reviewed by the study PI. All identifying participant data will be stored behind a VA firewall to ensure data security.

#### Statistical Methods

Aim 1 preliminary analyses will be conducted using established methods for qualitative research [58]. Debriefings will occur after each interview through a structured audio review and matrix displays. The debriefing format will highlight the content and key findings from each interview, enabling the study team to determine when saturation has been reached. The findings from these debriefings will be categorized into “rapid codes” on a matrix display signifying the key areas of the interview agenda they represent (eg, barriers to treatment, Deprexis content, and changes experienced in mood and functioning). Matrix displays create a visual representation of responses to key codes from each participant by lining up each primary response to a question side by side in a single row of a spreadsheet. Rapid codes are summarized into themes, revised continually, and used to determine saturation and inform intervention content areas. Subsequently, during formal analyses, all interviews will be recorded, transcribed verbatim, deidentified, and reviewed for accuracy against the recordings. Drawing from the literature, the interview agenda will be used to develop an initial coding structure that captures participant responses. An iterative process of transcript review will generate additional codes that will be added to the coding structure. The final coding structure will then be used by the research team to code and review all transcripts for concordance. The final codes will be entered into ATLAS.ti (ATLAS.ti Scientific Software Development GmbH) [59], a qualitative data management software tool, for review using applied thematic analysis. Applied thematic analysis is a systematic approach to coding, organizing, and interpreting qualitative data to best capture meaning via themes across participants’ responses; for example, within any given code, this approach captures the breadth of responses to create an overall theme. In accordance with the purpose of aim 1—to improve the further study and uptake of Deprexis in the RCT—we will look carefully for themes that describe the intended needs and desires of veterans, the perceived utility of Deprexis, changes noted in mood and functional domains, and other emergent themes. These themes will be used to identify several potentially modifiable components of Deprexis, which can be used to guide current and future study efforts.

For aim 2, linear mixed effects regression [60] with restricted maximum likelihood estimation will be used to model continuous outcomes (eg, changes in psychosocial functioning outcomes and depressive symptoms), and logistic models will be used to model changes in dichotomous outcomes (eg, significant change: ≥50% improvement in scores) over time. Linear mixed effects regression models will initially be fit using an interactive model (eg, treatment condition+time+treatment condition×time) and compared to an additive model (eg, treatment condition+time) using a likelihood ratio test. This nested comparison serves as a significance test of the treatment×time interaction and will be reported as such in the analyses [61]. *Condition* (treatment or TAU), *time* (before treatment, after treatment, and follow-up), and their interaction will be entered as fixed effects, and *participant* will be entered as a random effect (ie, random intercepts). Cohen *d* for the treatment×time interaction will be calculated using an effect size derived from the mean pretreatment-to-posttreatment and follow-up change in the treatment condition minus the mean pretreatment-to-posttreatment and follow-up change in the TAU control divided by the pooled pretreatment SD [62]. Simulations suggested that this effect size estimate was the most stable and robust for these types of treatment designs [63]. When comparisons are made within a time period, Welch 1-tailed *t* tests will be performed because they are ideal when the 2 samples have unequal variances or unequal sample sizes [64]. When comparisons are made within a treatment condition over time (repeated measures), a paired samples *t* test will be performed. As participants can engage in nonstudy mental health treatment, nonstudy treatment use will be included as a covariate in all analyses.

#### Methods for Additional Analyses

In an exploratory and hypothesis-generating mode, potential moderators for Deprexis treatment effects will be examined. Generalized linear mixed models with robust maximum likelihood estimation will be used to model continuous outcomes (eg, changes in psychosocial functioning outcomes and depressive symptoms), and logistic models will be used to model changes in dichotomous outcomes (eg, significant change: ≥50% improvement in scores) over time. *Participant* will be entered as a random effect (ie, random intercepts). Each potential moderator will be examined in a separate model for changes in primary outcomes (QIDS-SR-16, WHODAS-II, and SDS). Each model will include main effects for the potential moderator and time (before treatment, after treatment, and follow-up), 2-way interactions (moderator×time and time×treatment condition), and a 3-way interaction (moderator×time×treatment condition). To prevent spurious associations, the α value will be Bonferroni corrected [65]. A variable will be considered a moderator of treament effects if there is a significant 3-way interaction (moderator×time×treatment condition) after treatment or at 8-week follow-up.

#### Definition of Analysis Population Relating to Protocol Nonadherence and Any Statistical Methods to Handle Missing Data

Consistent with CONSORT (Consolidated Standards of Reporting Trials) recommendations [66], aim 2 analyses will be conducted across four groups: (1) the intention-to-treat group, which includes data from all randomized participants, regardless of treatment adherence or attrition; (2) the per-protocol group, which consists of participants who are considered treatment adherent, defined as ≥60 minutes of Deprexis use (which is consistent with previous research [29]); (3) the high-engagement group, which consists of participants who demonstrated strong treatment uptake, defined as ≥90 minutes of Deprexis use (this cutoff is approximately 1 SD below the mean for the treatment-adherent group in the general population Deprexis RCT); and (4) the completer group, which consists of participants who complete both baseline and posttreatment assessments. In the intent-to-treat group, the *Amelia II* (version 1.8.2) package in R [67] will be used to impute missing data with an expectation-maximization and bootstrapping algorithm. All available variables will be used to impute missing data, and the number of imputed datasets will be based on the proportion of missing data.

### Dissemination of Results

Several manuscripts are planned to align with the various study aims. In addition, results will be presented at research conferences, and summaries will be drafted for media releases. Author eligibility and order will be determined by the study PI.

### Oversight and Monitoring

#### Composition of the Data Monitoring Committee, Its Role, and Reporting Structure

The data safety monitoring board (DSMB) will be responsible for safeguarding the interests of trial participants, assessing the safety and efficacy of the interventions during the trial, and monitoring the overall conduct of the clinical trial [68]. The DSMB will provide recommendations about stopping or continuing the clinical trial. To contribute to enhancing the integrity of the trial, the DSMB may formulate recommendations relating to the selection, recruitment, and retention of participants; their management; improving adherence to protocol-specified regiments; and procedures for data management and quality control. The DSMB will consist of at least 3 members, all of whom are independent of the trial. Any protocol deviations or unanticipated problems involving risks to participants or others will be reported to the DSMB within 5 days, as well as to the IRB, and summarized in the study progress report and submitted during continuing review.

#### Interim Analyses

No interim analyses are planned.

#### Adverse Event Reporting and Harms

In cases of unanticipated problems involving risks to participants or others or apparent serious or continuing noncompliance with federal regulations for the protection of research participants or the requirements or determinations of the IRB, a written report will be prepared for submission to the central IRB and DSMB within 5 business days of notification. The report will include a brief narrative summary of the event as well as a determination of whether a causal relationship existed between the study procedures and the event, whether the informed consent should be revised because of the event, and whether all enrolled participants should be notified of the event. All team meetings will begin by asking whether any such events have occurred.

#### Frequency and Procedures for Auditing Trial Conduct

Yearly auditing will be conducted by an IRB member who is independent of the study and its investigators. This is to ensure that the study is compliant with the protocol and is meeting the standards of human participant research.

### Ethical Considerations

The protocol has been approved by the Central Texas Veterans Affairs Healthcare System IRB (protocol 2024-001). Any changes to the study protocol will be promptly communicated to relevant stakeholders.

A waiver of informed consent will be obtained for the completion of the eligibility screen. Informed consent will be obtained for the remainder of the study procedures. A watermarked paper copy of the informed consent will be included with the study recruitment letter. An electronic version of the consent form will be included in the Qualtrics eligibility survey. Veterans will be asked to review the consent form, and they will be asked whether they have any questions or concerns. Veterans who indicate that they have questions or concerns will be contacted by telephone by the research technician. Once all questions and concerns are responded to, the research technician will verbally ask whether the veteran is still interested in the study and if the veteran answers in the affirmative will send a link to DocuSign consent via encrypted email. Veterans who indicate that they do not have questions or concerns will be asked in the Qualtrics eligibility screen whether they are interested in participating in the study. If they answer in the affirmative, they will be sent a link to DocuSignconsent via encrypted email.

No participant data will be released in any form unless the investigators or study personnel are mandated to do so by law (eg, because of risk to self or others). All study data will be kept in accordance with VA guidelines, which require that electronic and paper data be kept for the duration of the study and for 6 fiscal years thereafter (for details on data storage and disposition, refer to VA Records Control Schedule 10-1 [69]). Participants will be assigned a study ID, which is linked to identifying information in a password-protected spreadsheet, which is stored behind the VA firewall. Study personnel who have access to study data will receive training in confidentiality policies and procedures.

All IRB-approved study team members may have access to participant-level data pending approval from the study PI. The full protocol will be available at the discretion of the study PI.

## Results

This study was funded in May 2024. Data collection is anticipated to take place between October 2024 and April 2029. Currently, 4 participants have been recruited into this study. The results are expected in November 2029.

## Discussion

### Summary

Research examining computer-delivered interventions in veterans lags behind that conducted in general population samples. This study will gather the qualitative experiences of veterans with a computerized intervention and will be the first RCT of a self-guided, computerized intervention for depressive symptoms and functional impairment in veterans. The results from the described study will demonstrate whether the positive results in general population trials of computerized interventions generalize to the veteran population. Furthermore, we broaden the target population of these types of interventions by including veterans with subthreshold symptoms of depression. The described trial has an explicit focus on functional outcomes because there is evidence that symptomatic recovery and functional improvements do not consistently improve in tandem after mental health interventions. Deprexis has previously been associated with significant improvement in disability and well-being; however, these were secondary outcomes. This study centralizes and significantly expands the assessment of psychosocial functioning across domains.

Depressive symptoms, independent of comorbid trauma symptoms, are an important driver of functional impairment and suicidality in veterans. When depressive symptoms co-occur with PTSD, these symptoms often persist after trauma-focused treatments. Thus, depressive symptoms should be an urgent target of intervention in veterans, even in the context of complex comorbid conditions. There is evidence that the depression treatment gap commonly described in community samples also exists for veterans who receive their care at VA facilities [20]. Various reasons for the existence of this treatment gap have been suggested, and it seems likely that multiple individual, logistical, and institutional barriers may prevent veterans with depressive symptoms from receiving appropriate care. Cost-effective, accessible computerized interventions would broaden the reach of depressive treatments available within the VA system.

Importantly, the VA system is an appropriate site for testing an intervention such as Deprexis. The broad screening for depressive symptoms that occurs at all VA clinics (ie, outside of specialty mental health clinics) and the integrated mental health record allow for the timely identification of veterans presenting with relatively mild depressive symptoms. There is an opportunity to prevent chronicity and disease progression by focusing treatment on mild to moderate depressive symptoms. There is evidence that Deprexis may be especially effective for individuals with lower depressive symptom severity [53]. Our long-term vision is that brief, computerized interventions such as Deprexis can be integrated in a stepped-care approach, providing rapid intervention for veterans with mild to moderate depressive symptoms, while reserving resource-intensive treatments such as face-to-face psychotherapy for individuals with severe symptomatology.

Alongside the aforementioned strengths, this study has several limitations. The choice of a TAU control group is a relatively weak comparator. Follow-up studies comparing Deprexis to active treatment conditions would provide further evidence for the relative impact of the intervention. All assessments administered in this study are self-report questionnaires, which are prone to certain types of bias (eg, desirability bias). If the efficacy of Deprexis is supported in this trial, examining whether change also generalizes to other assessments, such as ecological momentary assessment or tracking, may be merited. Relatedly, because no clinical interviews are conducted, we are not able to assess the presence or absence of clinical diagnoses. Finally, use will be closely monitored in this trial, which may not be feasible in clinical settings. Automating use reminders may be an important step to promote successful uptake in the VA system.

### Conclusions

Despite the aforementioned limitations, we believe that the described study will provide much needed initial evidence for the effectiveness of computerized interventions broadly and Deprexis specifically for veterans presenting with depressive symptoms and functional impairment. The results obtained in this study could inform the rollout of targeted computer-delivered interventions that address common barriers to mental health care in veterans. This is especially pertinent given that demand for VA behavioral health care outpaces supply, resulting in increased outsourcing of behavioral health care in the form of community care referrals [70]. Building a diverse arsenal of psychosocial interventions, including computerized interventions, potentially allows more veterans to receive behavioral health care within the VA system. Furthermore, giving veterans additional mental health care options could improve overall engagement with treatment [71]. We believe that the results of this study could potentially extend and advance routine care for veterans with depressive symptoms, which is an essential step toward closing the treatment gap and providing the highest quality care for veterans with complex mental health needs.

## Abbreviations

BDI-II: Beck Depression Inventory–Second Edition
CONSORT: Consolidated Standards of Reporting Trials
DSMB: data safety monitoring board
IIP-SC: Inventory of Interpersonal Problems–Short Circumplex Form
IRB: institutional review board
ISEL-SF: Interpersonal Support Evaluation List–Short Form
MDD: major depressive disorder
PCL-5: posttraumatic stress disorder Checklist for Diagnostic and Statistical Manual of Mental Disorders, Fifth Edition
PDSQ: Psychiatric Diagnostic Screening Questionnaire
PHQ: Patient Health Questionnaire
PI: principal investigator
PTSD: posttraumatic stress disorder
QIDS-SR: Quick Inventory of Depressive Symptomatology–Self-Report
QLS: Quality of Life Scale
RCT: randomized controlled trial
SDS: Sheehan Disability Scale
TAU: treatment-as-usual
VA: US Department of Veterans Affairs
WHODAS-II: World Health Organization Disability Assessment Schedule II

## Disclaimer

The contents of this manuscript do not represent the views of the US Department of Veterans Affairs or the United States government. As RP, JM, JB, PNP, EP, and SKC are employees of the US government and contributed to this paper as part of their official duties, the work is not subject to US copyright.

## Data Availability

A deidentified participant-level dataset may be available to the scientific community pending appropriate approvals. The full statistical code will be available at the discretion of the principal investigator.

## References

[1] (2004). The global burden of disease: 2004 update. World Health Organization (WHO).

[2] Osby U, Brandt L, Correia N, Ekbom A, Sparén P (2001). Excess mortality in bipolar and unipolar disorder in Sweden. Arch Gen Psychiatry.

[3] Broadhead WE, Blazer DG, George LK, Tse CK (1990). Depression, disability days, and days lost from work in a prospective epidemiologic survey. JAMA.

[4] Pyne JM, Patterson TL, Kaplan RM, Gillin JC, Koch WL, Grant I (1997). Assessment of the quality of life of patients with major depression. Psychiatr Serv.

[5] Choi S, Lee S, Matejkowski J, Baek YM (2014). The relationships among depression, physical health conditions and healthcare expenditures for younger and older Americans. J Ment Health.

[6] Liu Y, Collins C, Wang K, Xie X, Bie R (2019). The prevalence and trend of depression among veterans in the United States. J Affect Disord.

[7] Curry JF, Aubuchon-Endsley N, Brancu M, Runnals JJ, Fairbank JA, VA Mid-Atlantic Mirecc Women Veterans Research Workgroup, VA Mid-Atlantic Mirecc Registry Workgroup (2014). Lifetime major depression and comorbid disorders among current-era women veterans. J Affect Disord.

[8] Maguen S, Griffin BJ, Copeland LA, Perkins DF, Richardson CB, Finley EP, Vogt D (2022). Trajectories of functioning in a population-based sample of veterans: contributions of moral injury, PTSD, and depression. Psychol Med.

[9] VA national suicide data report 2005-2015. Office of Mental Health and Suicide Prevention.

[10] Ilgen MA, Bohnert AS, Ignacio RV, McCarthy JF, Valenstein MM, Kim HM, Blow FC (2010). Psychiatric diagnoses and risk of suicide in veterans. Arch Gen Psychiatry.

[11] Nichter B, Norman S, Haller M, Pietrzak RH (2019). Psychological burden of PTSD, depression, and their comorbidity in the U.S. veteran population: suicidality, functioning, and service utilization. J Affect Disord.

[12] Cuijpers P, de Graaf R, van Dorsselaer S (2004). Minor depression: risk profiles, functional disability, health care use and risk of developing major depression. J Affect Disord.

[13] Cuijpers P, Smit F (2004). Subthreshold depression as a risk indicator for major depressive disorder: a systematic review of prospective studies. Acta Psychiatr Scand.

[14] Fergusson DM, Horwood LJ, Ridder EM, Beautrais AL (2005). Subthreshold depression in adolescence and mental health outcomes in adulthood. Arch Gen Psychiatry.

[15] Hardeveld F, Spijker J, de Graaf GR, Nolen WA, Beekman AT (2010). Prevalence and predictors of recurrence of major depressive disorder in the adult population. Acta Psychiatr Scand.

[16] Cuijpers P, Smit F, van Straten A (2007). Psychological treatments of subthreshold depression: a meta-analytic review. Acta Psychiatr Scand.

[17] Wells KB, Hays RD, Burnam MA, Rogers W, Greenfield S, Ware JE (1989). Detection of depressive disorder for patients receiving prepaid or fee-for-service care. Results from the Medical Outcomes Study. JAMA.

[18] van Schaik DJ, Klijn AF, van Hout HP, van Marwijk HW, Beekman A, de Haan M, van Dyck R (2004). Patients' preferences in the treatment of depressive disorder in primary care. Gen Hosp Psychiatry.

[19] Farmer MM, Rubenstein LV, Sherbourne CD, Huynh A, Chu K, Lam C, Fickel JJ, Lee ML, Metzger ME, Verchinina L, Post EP, Chaney EF (2016). Depression quality of care: measuring quality over time using VA electronic medical record data. J Gen Intern Med.

[20] Funderburk JS, Sugarman DE, Labbe AK, Rodrigues A, Maisto SA, Nelson B (2011). Behavioral health interventions being implemented in a VA primary care system. J Clin Psychol Med Settings.

[21] Hoge CW, Castro CA, Messer SC, McGurk D, Cotting DI, Koffman RL (2004). Combat duty in Iraq and Afghanistan, mental health problems, and barriers to care. N Engl J Med.

[22] Grubaugh AL, Gros KS, Davidson TM, Frueh BC, Ruggiero KJ (2014). Providers’ perspectives regarding the feasibility and utility of an internet-based mental health intervention for veterans. Psychol Trauma Theo Res Pract Pol.

[23] Brown M, Glendenning A, Hoon AE, John A (2016). Effectiveness of web-delivered acceptance and commitment therapy in relation to mental health and well-being: a systematic review and meta-analysis. J Med Internet Res.

[24] Andrews G, Basu A, Cuijpers P, Craske MG, McEvoy P, English CL, Newby JM (2018). Computer therapy for the anxiety and depression disorders is effective, acceptable and practical health care: an updated meta-analysis. J Anxiety Disord.

[25] Karyotaki E, Riper H, Twisk J, Hoogendoorn A, Kleiboer A, Mira A, Mackinnon A, Meyer B, Botella C, Littlewood E, Andersson G, Christensen H, Klein JP, Schröder J, Bretón-López J, Scheider J, Griffiths K, Farrer L, Huibers MJ, Phillips R, Gilbody S, Moritz S, Berger T, Pop V, Spek V, Cuijpers P (2017). Efficacy of self-guided internet-based cognitive behavioral therapy in the treatment of depressive symptoms: a meta-analysis of individual participant data. JAMA Psychiatry.

[26] Spijkerman MP, Pots WT, Bohlmeijer ET (2016). Effectiveness of online mindfulness-based interventions in improving mental health: a review and meta-analysis of randomised controlled trials. Clin Psychol Rev.

[27] Meyer B, Berger T, Caspar F, Beevers CG, Andersson G, Weiss M (2009). Effectiveness of a novel integrative online treatment for depression (Deprexis): randomized controlled trial. J Med Internet Res.

[28] Digital therapy that works. Deprexis.

[29] Beevers CG, Pearson R, Hoffman JS, Foulser AA, Shumake J, Meyer B (2017). Effectiveness of an internet intervention (Deprexis) for depression in a United States adult sample: a parallel-group pragmatic randomized controlled trial. J Consult Clin Psychol.

[30] Home page. Quatlrics.

[31] Rush AJ, Trivedi MH, Ibrahim HM, Carmody TJ, Arnow B, Klein DN, Markowitz JC, Ninan PT, Kornstein S, Manber R, Thase ME, Kocsis JH, Keller MB (2003). The 16-item quick inventory of depressive symptomatology (QIDS), clinician rating (QIDS-C), and self-report (QIDS-SR): a psychometric evaluation in patients with chronic major depression. Biol Psychiatry.

[32] Zimmerman M, Mattia JI (2001). The psychiatric diagnostic screening questionnaire: development, reliability and validity. Compr Psychiatry.

[33] Hirschfeld RM, Williams JB, Spitzer RL, Calabrese JR, Flynn L, Keck Jr PE, Lewis L, McElroy SL, Post RM, Rapport DJ, Russell JM, Sachs GS, Zajecka J (2000). Development and validation of a screening instrument for bipolar spectrum disorder: the Mood Disorder Questionnaire. Am J Psychiatry.

[34] Beck AT, Steer RA, Brown GK (1996). Beck depression inventory (BDI-II). American Psychological Association.

[35] Üstün TB, Kostanjsek N, Chatterji S, Rehm J (2010). Measuring health and disability: manual for WHO disability assessment schedule (‎WHODAS 2.0)‎. World Health Organization (WHO).

[36] Sheehan D (1986). The Anxiety Disease: New Hope for the Millions Who Suffer from Anxiety.

[37] Arbuckle R, Frye MA, Brecher M, Paulsson B, Rajagopalan K, Palmer S, Degl' Innocenti A (2009). The psychometric validation of the Sheehan Disability Scale (SDS) in patients with bipolar disorder. Psychiatry Res.

[38] Sheehan KH, Sheehan DV (2008). Assessing treatment effects in clinical trials with the Discan metric of the Sheehan Disability Scale. Int Clin Psychopharmacol.

[39] von Glischinski M, von Brachel R, Hirschfeld G (2019). How depressed is "depressed"? A systematic review and diagnostic meta-analysis of optimal cut points for the Beck Depression Inventory revised (BDI-II). Qual Life Res.

[40] Sprinkle SD, Lurie D, Insko SL, Atkinson G, Jones GL, Logan AR, Bissada NN (2002). Criterion validity, severity cut scores, and test-retest reliability of the Beck Depression Inventory-II in a university counseling center sample. J Couns Psychol.

[41] Soldz S, Budman S, Demby A, Merry J (1995). Inventory of interpersonal problems—short circumplex form (IIP-SC). APA PsycTests.

[42] Barkham M, Hardy GE, Startup M (1996). The IIP-32: a short version of the inventory of interpersonal problems. Br J Clin Psychol.

[43] Cohen S, Mermelstein R, Kamarck T, Hoberman H, Sarason IG, Sarason BR (1985). Measuring the functional components of social support. Social Support: Theory, Research and Applications.

[44] Frenette É, Ouellet MC, Guay S, Lebel J, Békés V, Belleville G (2023). The effect of an internet-based cognitive behavioral therapy intervention on social support in disaster evacuees. J Clin Psychol.

[45] Payne TJ, Andrew M, Butler KR, Wyatt SB, Dubbert PM, Mosley TH (2012). Psychometric evaluation of the interpersonal support evaluation list–short form in the ARIC study cohort. SAGE Open.

[46] Flanagan JC (1982). Measurement of quality of life: current state of the art. Arch Phys Med Rehabil.

[47] Burckhardt CS, Anderson KL (2003). The Quality of Life Scale (QOLS): reliability, validity, and utilization. Health Qual Life Outcomes.

[48] Burckhardt CS, Clark SR, Bennett RM (1993). Fibromyalgia and quality of life: a comparative analysis. J Rheumatol.

[49] Blevins CS, Weathers FW, Davis MT, Witte TK, Domino JL (2015). The posttraumatic stress disorder checklist for DSM-5 (PCL-5): development and initial psychometric evaluation. J Trauma Stress.

[50] Wortmann JH, Jordan AH, Weathers FW, Resick PA, Dondanville KA, Hall-Clark B, Foa EB, Young-McCaughan S, Yarvis JS, Hembree EA, Mintz J, Peterson AL, Litz BT (2016). Psychometric analysis of the PTSD Checklist-5 (PCL-5) among treatment-seeking military service members. Psychol Assess.

[51] Borkovec TD, Nau SD (1972). Credibility of analogue therapy rationales. J Behav Ther Exp Psychiatry.

[52] Devilly GJ, Borkovec TD (2000). Psychometric properties of the credibility/expectancy questionnaire. J Behav Ther Exp Psychiatry.

[53] Pearson R, Pisner D, Meyer B, Shumake J, Beevers C (2019). A machine learning ensemble to predict treatment outcomes following an internet intervention for depression. Psychol Med.

[54] Attkisson CC, Greenfield TK, Munish ME (2004). The UCSF client satisfaction scales: I. The client satisfaction questionnaire-8. Use Psychol Test Treat Plan Outcomes Assess Instrum Adults. Volume 3.

[55] Attkisson CC, Zwick R (1982). The client satisfaction questionnaire. Psychometric properties and correlations with service utilization and psychotherapy outcome. Eval Program Plann.

[56] Fusch P, Ness L (2015). Are we there yet? Data saturation in qualitative research. Qual Rep.

[57] Hundt NE, Barrera TL, Robinson A, Cully JA (2014). A systematic review of cognitive behavioral therapy for depression in veterans. Mil Med.

[58] Hamilton AB, Finley EP (2019). Qualitative methods in implementation research: an introduction. Psychiatry Res.

[59] Germany: scientific software development GmbH. Atlas.ti.

[60] Zhang X A tutorial on restricted maximum likelihood estimation in linear regression and linear mixed-effects model. A*STAR-NUS Clinical Imaging Research Center.

[61] Lewis F, Butler A, Gilbert L (2010). A unified approach to model selection using the likelihood ratio test. Methods Ecol Evol.

[62] McGough JJ, Faraone SV (2009). Estimating the size of treatment effects: moving beyond *P* values. Psychiatry (Edgmont).

[63] Morris SB (2007). Estimating effect sizes from pretest-posttest-control group designs. Organ Res Methods.

[64] Delacre M, Lakens D, Leys C (2017). Why psychologists should by default use Welch’s t-test instead of Student’s t-test. Int Rev Soc Psychol.

[65] Sedgwick P (2012). Multiple significance tests: the Bonferroni correction. BMJ.

[66] Bennett JA (2005). The consolidated standards of reporting trials (CONSORT): guidelines for reporting randomized trials. Nurs Res.

[67] Honaker J, King G, Blackwell M Package ‘Amelia’. Cran R.

[68] Holbein B, Rape MT, Hammack BN, Melvin A, Reider C, Knox TA (2021). Institutionally chartered data and safety monitoring boards: structured approaches to assuring participant safety in clinical research. J Investig Med.

[69] (2021). Records control schedule 10-1. Department of Veterans Affairs (US).

[70] Vanneman ME, Rosen AK, Wagner TH, Shwartz M, Gordon SH, Greenberg G, Zheng T, Cook J, Beilstein-Wedel E, Greene T, Kelley AT (2023). Differences between VHA-delivered and VHA-purchased behavioral health care in service and patient characteristics. Psychiatr Serv.

[71] Karlin BE, Brenner LA (2020). Improving engagement in evidence‐based psychological treatments among veterans: direct‐to‐consumer outreach and pretreatment shared decision‐making. Clin Psychol Sci Pract.

